# Bravery in the Ranks: the Work of the Overseas Medical Corps

**Published:** 1915-07-10

**Authors:** 


### BRAVERY IN THE RANKS.

### The Work of the Overseas Medical Corps.

Some splendid examples of heroism on the part of the Medical Service are recorded in the Special Supplement of the *London Gazette* which was issued last week, containing particulars of the deeds which had gained for nearly 600 persons the Distinguished Conduct Medal.

Sergeant T. M. Brown, of the No. 1 Field Ambulance of the Canadian Army Medical Corps, was rewarded for conspicuous gallantry and devotion to duty on the night of April 22-23. Being cut off from the collecting station by shell fire, he remained with the wounded near the trenches all day, and then brought all of them in safety to the station on the following night. Sergeant T. B. Carter, of the 19th Field Ambulance of the R.A.M.C., at Houplines tended the wounded under heavy fire, being often continuously on duty by day and night. "This officer," the official report adds, "has previously displayed great gallantry in assisting the wounded under fire." Private Cartwright, of the 14th Field Ambulance of the R.A.M.C., is commended in the *Gazette* "for conspicuous devotion to duty since August 1914 in tending the wounded, frequently under fire. On February 25 he was reported for most heroic conduct under shell fire."

Sergeant-Major A. E. Clifton, of the Canadian Army Medical Corps, displayed conspicuous gallantry, coolness, and devotion to duty while under fire at Vlamertinghe on April 27, when he assisted to remove some wounded from the church to a place of safety. Third-class Assistant Surgeon K. P. Elloy, of the No. 7 British Field Ambulance, Indian Subordinate Medical Department, gained his medal in circumstances which are best described in the bald, unvarnished words of the *Gazette*: "For conspicuous gallantry and devotion to duty near Ypres during the operations from April 24 to May 4 in collecting and treating wounded under heavy fire, and especially at St. Jean on April 29, when, on the advanced dressing station being destroyed by shell fire, he helped to remove the wounded to a place of safety, displaying great coolness and resource."

### Striking Cases.

Of Private W. Herd, of the R.A.M.C. (T.F.), it is recorded that he left his trench and went to the rear across the open at great personal risk in order to obtain some morphia for a wounded man.

We now pass on to give the following particularly striking case—that of A. J. Hook, a driver in the No. 4 Motor Ambulance Convoy of the British Red Cross Society. He is rewarded for conspicuous gallantry and devotion to duty while returning from a dressing station on April 21 when he carried a wounded man on his back for a mile to the nearest ambulance waggon, his own waggon having broken down and the road being under heavy shell fire at the time.

Next, the record of Private A. W. Howitt, of the R.A.M.C., speaks for itself. "For conspicuous gallantry and devotion to duty from December to March when in charge of an ambulance waggon. On more than one occasion he has volunteered for exceptionally dangerous duty, and during an action at Neuve Chapelle in October was able to bring away two waggon-loads after the troops had withdrawn from their positions."

The remaining instances in this category include that of Sergeant C. Ingram, of the 84th Field Ambulance of the R.A.M.C. (T.F.), who obtained his medal for conspicuous gallantry and devotion to duty in visiting the trenches and removing the wounded under heavy fire on February 28 and March 4. He was severely wounded on March 5.

Of Sergeant F. Newman, of the R.A.M.C., the *London Gazette* says that he received his medal "For conspicuous coolness and gallantry when in charge of advanced dressing stations. In the presence of danger this officer has carried out his duties with great ability." Private F. Turner, of the Canadian Army Medical Corps, is commended for conspicuous gallantry and devotion to duty when attending to wounded at Wieltje. When the dressing station had been heavily shelled he carried his wounded to a neighbouring ditch and continued to tend them under heavy shell fire for the greater part of the day, remaining until all had been removed. A high tribute is paid to Sergeant H. J. V. Voisey, of the R.A.M.C., who "has displayed great coolness when collecting wounded at night, and has shown excellent initiative on many occasions under fire. He performed very good work at the dressing station at Vailly in September under very trying circumstances." Finally, Corporal R. J. Wood, of the 1st Wessex Field Ambulance, R.A.M.C. (T.F.), was decorated for gallantry while in command of stretcher-bearers under heavy fire at Neuve Chapelle.

### WORK AT THE DARDANELLES.

During the week-end a further Supplement to the *London Gazette* was published containing particulars of gallant deeds performed at the Dardanelles, and again the names of Army Medical Corps men are prominent.

Private L. W. Burnett, of the Australian Army Medical Corps; Staff-Sergeant H. Jackson, of the same corps; and Lance-Corporal W. Singleton, of the New Zealand Field Ambulance, are all rewarded "for exceptionally gallant work and devotion to duty under heavy fire near Kaba Tepe."

During the same devoted operations Lance-Corporal G. Steedman, of the New Zealand Medical Corps, rescued a wounded man under fire on April 27, and two days later attended two wounded men under still heavier fire; while Private T. Stockdill, stretcher-bearer to the Canterbury Battalion of the New Zealand Forces, recovered wounded men on the open beach under heavy fire.

Corporal J. W. Jones and Private A. Cook, of the R.A.M.C. (T.F.), gained their medals for conspicuous bravery during the landing operations at Cape Helles in attending wounded and supplying them with water under heavy fire.

It only remains to add how very gratifying it is to note in these lists the large share of praise gained by the bravery of the men in the various Medical Corps which our Dominions have furnished to the Empire.

